# Association between insurance status and in‐hospital outcomes in patients with out‐of‐hospital ventricular fibrillation arrest

**DOI:** 10.1002/clc.23564

**Published:** 2021-03-04

**Authors:** Samir B. Pancholy, Gaurav A. Patel, Dhara D. Patel, Neil Patel, Shivam A. Pancholy, Purveshkumar Patel, Linda Thomas‐Hemak, Tejas M. Patel, David J. Callans

**Affiliations:** ^1^ Division of Cardiology, Department of Medicine The Wright Center for Graduate Medical Education Scranton Pennsylvania USA; ^2^ Department of Cardiology Apex Heart Institute Ahmedabad India; ^3^ Perelman School of Medicine University of Pennsylvania Philadelphia Pennsylvania USA

**Keywords:** mortality, uninsured, ventricular fibrillation

## Abstract

**Background:**

Lack of health insurance is associated with adverse clinical outcomes; however, the association between health insurance status and in‐hospital outcomes after out‐of‐hospital ventricular fibrillation (OHVFA) arrest is unclear.

**Hypothesis:**

Lack of health insurance is associated with worse in‐hospital outcomes after out‐of‐hospital ventricular fibrillation arrest.

**Methods:**

From January 2003 to December 2014, hospitalizations with a primary diagnosis of OHVFA in patients ≥18 years of age were extracted from the Nationwide Inpatient Sample. Patients were categorized into insured and uninsured groups based on their documented health insurance status. Study outcome measures were in‐hospital mortality, utilization of implantable cardioverter defibrillator (ICD), and cost of hospitalization. Inverse probability weighting adjusted binary logistic regression was performed to identify independent predictors of in‐hospital mortality and ICD utilization and linear regression was performed to identify independent predictors of cost of hospitalization.

**Results:**

Of 188 946 patients included in the final analyses, 178 005 (94.2%) patients were insured and 10 941 (5.8%) patients were uninsured. Unadjusted in‐hospital mortality was higher (61.7% vs. 54.7%, *p* < .001) and ICD utilization was lower (15.3% vs. 18.3%, *p* < .001) in the uninsured patients. Lack of health insurance was independently associated with higher in‐hospital mortality (O.R = 1.53, 95% C.I. [1.46–1.61]; *p* < .001) and lower utilization of ICD (O.R = 0.84, 95% C.I [0.79–0.90], *p* < .001). Cost of hospitalization was significantly higher in uninsured patients (median [interquartile range], *p*‐value) ($) (39 650 [18 034‐93 399] vs. 35 965 [14 568.50‐96 163], *p* < .001).

**Conclusion:**

Lack of health insurance is associated with higher in‐hospital mortality, lower utilization of ICD and higher cost of hospitalization after OHVFA.

## 1 INTRODUCTION

Cardiac arrest is the leading cause of cardiac mortality annually in the United States.^1^ Etiologies of cardiac arrest are heterogeneous, usually categorized into asystole, pulseless electrical activity, and ventricular tachycardia (VT)/ventricular fibrillation (VF). Cardiac arrest due to asystole and pulseless electrical activity are highly variable in true cause (i.e. cardiac vs. non‐cardiac causality); we chose to focus our analysis on cardiac arrest with documented VT/VF as this condition is more homogenous and outcomes are better.

Lack of health insurance and low socioeconomic status are associated with poor health outcomes, likely due to compromised access to preventative and routine care.^2^, ^3^ Further, disparities in healthcare access and utilization exist, which likely contributes to higher incidence of cardiac arrest in individuals of lower socio‐economic status.^4^, ^5^ The association between health insurance status and in hospital outcomes after ST‐segment elevation myocardial infarction have been studied previously with a demonstrated, independent association found between outcomes disadvantage and uninsured status.^6^ The effect of health insurance status on outcome of cardiac arrest remains unclear.

We sought to evaluate the independent effect of health insurance status on in‐hospital mortality, implantable cardioverter defibrillator (ICD) utilization and cost of care in patients presenting with out‐of‐hospital VF arrest (OHVFA) using a large national hospital database.

## 2 METHODS

### 2.1 The nationwide inpatient sample database

The nationwide inpatient sample (NIS) database is the largest all‐payer database in the United States managed by the Agency for Healthcare Research and Quality Healthcare and Cost and Utilization Project. The NIS records information on 20% stratified sample of hospital discharges of all community and non‐Federal United States hospitals prior to 2012.^7^ Since 2012, the NIS has been significantly redesigned and represents 20% stratified sample of all discharges of the US hospitals.^8^ The database contains information on patients' demographic, such as age, gender, race, household income category, and primary payer status, as well as all diagnoses and procedural information in the form of International Classification of Diseases, Ninth Revision, Clinical Modification (ICD‐9‐CM) and Clinical Classification Software codes. The database provides discharge weights to calculate regional and national estimates. The study was reviewed by the local institutional review board and was deemed retrospective and was given clearance.

### 2.2 Study population

**FIGURE 1:**
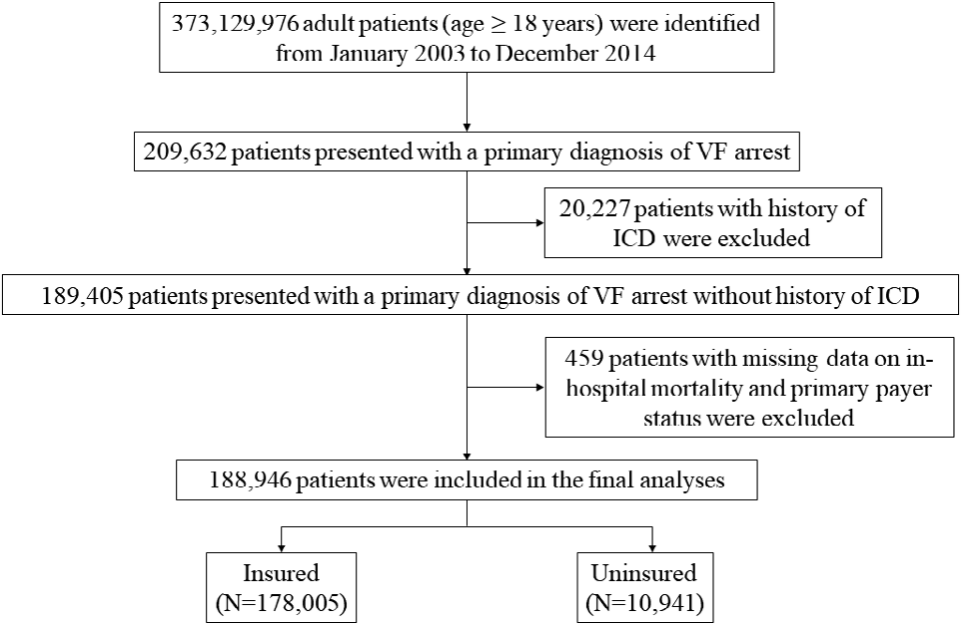
Data extraction and patient selection methods. Detailed demonstration of data extraction and patient selection methods

From January 2003 to December 2014, hospitalizations with a primary diagnosis of VF arrest in patients 18 years of age and older were extracted by searching for the ICD‐9‐CM codes for VF (427.4, 427.41, 427.42, and 427.5). Patients with missing data on primary payer status and in‐patient mortality were excluded from the final analyses. Figure 1 demonstrates data extraction and patient selection methods.

The primary payer status in the NIS database has been categorized as Medicare, Medicaid, private insurance, self‐pay, no charge, and other insurance. If the primary payer status indicated self‐pay or no charge, those patients were considered to be uninsured. Patients' baseline comorbidities and procedural characteristics were extracted using ICD‐9‐CM and CCS codes (Supplemental Table 1). Charlson comorbidity variables were identified based on Elixhauser methods using standard AHRQ variables.^9^

### 2.3 Statistical analyses

Weighted sample was used for all analyses. All statistical analyses were performed using IBM SPSS Statistics, version 26 (IBM Corporation, Armonk, NY). Categorical variables were expressed as proportions, whereas central tendencies of continuous variables were expressed as means or medians, as appropriate. Continuous variables were tested for normality using Kolmogorov–Smirnov test. Baseline and hospital characteristics of patients were compared using Pearson Chi‐square test for categorical variables and Mann–Whitney *U* test for continuous variables due to non‐normal distribution. A 2‐sided *p* value of < .05 was considered statistically significant.

Age, gender, race, prior stroke, diabetes, hypertension, atrial arrhythmias (atrial fibrillation or atrial flutter), chronic kidney disease (CKD), valvular heart disease, long‐term use of anti‐coagulants, smoking, alcoholism, drug abuse, congestive heart failure, peripheral vascular disease, previous myocardial infarction (MI), previous coronary revascularization with either percutaneous coronary intervention (PCI) or coronary artery bypass graft (CABG) surgery, overweight status, obesity, morbid obesity, and income status were included in the binary logistic regression analysis used to derive probability value for each hospital discharge used as the propensity score. We performed inverse probability weighted analysis using binary logistic regression adjusting for propensity score and distal variables to identify the independent predictors of in‐hospital mortality. We also sought to analyze the independent predictors of ICD prescription and assess the effect of insurance status on ICD utilization using a similar method. Age, gender, previous MI, previous coronary revascularization, and income status were used in the binary logistic regression to derive probability value for individual hospitalizations. Inverse probability weighting adjusted binary logistic regression was then performed to identify the independent predictors of ICD insertion. Receiver operating characteristic (ROC) derived area under the curve (AUC) was used to evaluate the statistical significance of the model.

## 3 RESULTS

From January 2003 to December 2014, a total of 446 348 443 patients were hospitalized in the United States, of which 373 129 976 patients ≥18 years of age were extracted. 209 632 patients presented with a primary diagnosis of VF arrest, of which 20 227 patients had history of ICD insertion in the past and were excluded. Of 189 405 patients, 459 patients with missing data on in‐hospital mortality and primary payer status were excluded. A total of 188 946 patients were included in the final analyses, of which 178 005 (94.2%) patients were insured and 10 941 (5.8%) patients were uninsured.

**FIGURE 2:**
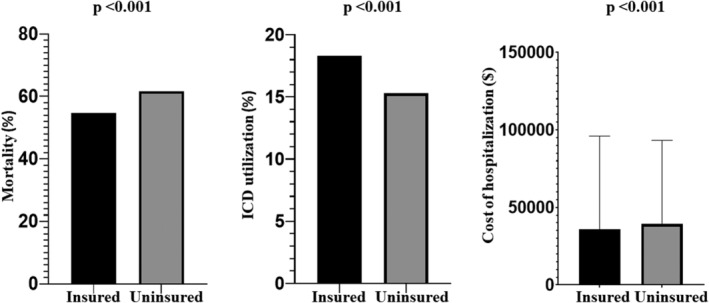
Comparative in‐hospital outcomes in patients with and without insurance. Lack of health insurance is associated with a higher in‐hospital mortality, lower utilization of internal cardioverter defibrillator (ICD) for secondary prevention and higher cost of hospitalization

Table 1 depicts baseline patient and hospital characteristics of the study population. Patients who were uninsured were significantly younger and were more frequently male. History of previous stroke, hypertension, diabetes, congestive heart failure, peripheral vascular diseases, atrial arrhythmias, CKD, previous MI, previous coronary revascularization, oral anticoagulant therapy use, and valvular heart diseases were all significantly lower in the uninsured patients. Compared to insured patients, uninsured patients had higher incidence of smoking, alcoholism, drug abuse, and obesity. Uninsured patients were more likely to be in low household income category and had significantly lower Charlson comorbidity index. In‐hospital outcomes of the study population are depicted in Table 2. Cardiogenic shock, acute stroke, gastrointestinal (GI) bleeding, and acute kidney injury (AKI) were significantly higher in the uninsured patients. Significantly higher proportion of uninsured patients left the hospital against medical advice (AMA) compared to insured patients (0.6% vs. 0.3%, *p* < .001). In‐hospital mortality was significantly higher in the uninsured patients (61.7% vs. 54.7%, *p* < .001) (Figure 2). Secondary prevention ICD utilization was significantly lower in the uninsured patients compared to insured patients (18.3% vs. 15.3%, *p* < .001) (Figure 2).

**TABLE 1:** Baseline patient and hospital characteristics of study population

Characteristics	Insured (*n* = 177 948)	Uninsured (*n* = 10 941)	*p*‐value
Age (years) (Median [interquartile range])	68.0 (57.0–78.0)	52.0 (43.0–60.0)	<.001
Male	104 371 (58.7%)	6892 (63.1%)	<.001
Female	73 577 (41.3%)	4039 (36.9%)	
White	106 221 (72.9%)	5674 (60.2%)	<.001
Black	21 587 (14.8%)	1738 (14.8%)	
Hispanic	9601 (6.6%)	1189 (12.6%)	
Asian or Pacific Islander	3354 (2.3%)	222 (2.4%)	
Native American	956 (0.7%)	46 (0.5%)	
Other	4082 (2.8%)	561 (5.9%)	
Median household income category^a^			
Below median national income category	98 921 (55.6%)	6957 (63.6%)	<.001
Above median national income category	79 084 (44.4%)	3984 (36.4%)	
Comorbidities			
Prior stroke	3565 (2.0%)	101 (0.9%)	<.001
Hypertension	97 666 (54.9%)	4257 (38.9%)	<.001
Congestive heart failure	59 397 (33.4%)	2180 (19.9%)	<.001
Diabetes	58 896 (33.1%)	2662 (24.3%)	<.001
Atrial fibrillation/flutter	42 196 (23.7%)	1463 (13.4%)	<.001
Chronic kidney disease	31 024 (17.4%)	679 (6.2%)	<.001
Peripheral vascular disease	13 733 (7.7%)	301 (2.8%)	<.001
Previous myocardial infarction	18 165 (10.2%)	743 (6.8%)	<.001
Previous revascularization	26 223 (14.7%)	766 (7.0%)	<.001
Valvular heart disease	2064 (1.2%)	33 (0.3%)	<.001
Long‐term use of anticoagulants	8836 (5%)	249 (2.3%)	<.001
Body mass index 25–29.9 kg/m^2^	247 (0.1%)	30 (0.3%)	<.001
Body mass index 30–39.9 kg/m^2^	8702 (4.9%)	645 (5.9%)	<.001
Body mass index ≥40 kg/m^2^	7161 (4.0%)	448 (4.1%)	.700
Smoking	32 896 (18.5%)	2989 (27.3%)	<.001
Alcoholism	9247 (5.2%)	1859 (17.0%)	<.001
Drug abuse	24 614 (13.8%)	3716 (34.0%)	<.001
Charlson comorbidity index [Median (interquartile range)]	2.0 (1.0–4.0)	2.0 (0.0–3.0)	<.001
Hospital location			
Urban	100 441 (88.8%)	6205 (92.0%)	<.001
Rural	12 708 (11.2%)	538 (8.0%)	
Teaching status of the hospitals			
Nonteaching	60 380 (53.4%)	3516 (52.1%)	<.001
Teaching	52 769 (46.6%)	3227 (47.9%)	
Hospital size			<.001
Small	18 783 (11.4%)	986 (9.7%)	
Medium	41 206 (25.1%)	2545 (25.2%)	
Large	104 330 (63.5%)	6587 (65.1%)	
Hospital region			<.001
Northeast	28 945 (17.5%)	1169 (11.5%)	
Midwest	40 783 (24.7%)	2079 (20.4%)	
South	59 041 (35.8%)	4748 (46.6%)	
West	36 311 (22%)	2191 (21.5%)	

^a^ This represents a quartile classification of the estimated median household income of residents in the patient's zip code.

**TABLE 2:** In‐hospital outcomes of the study population

Characteristics	Insured (*n* = 177 948)	Uninsured (*n* = 10 941)	*p*‐value
Sepsis	9148 (5.1%)	628 (5.7%)	.006
Cardiogenic shock	16 970 (9.5%)	1280 (11.7%)	<.001
Acute cerebrovascular accident	4255 (2.4%)	310 (2.8%)	.003
Gastrointestinal bleeding	7012 (3.9%)	753 (6.9%)	<.001
Acute kidney injury	41 038 (23.12%)	2975 (27.2%)	<.001
In‐hospital revascularization	8047 (4.5%)	496 (4.5%)	.950
Mechanical circulatory support	4253 (2.4%)	330 (3.0%)	<.001
Mechanical ventilation	117 491 (66%)	8106 (74.1%)	<.001
Implantable cardioverter defibrillator utilization	32 519 (18.3%)	1672 (15.3%)	<.001
Length of hospitalization (days) (Median (interquartile range])	3 (1–7)	2 (1–6)	<.001
Total charges ($) (Median [interquartile range])	35 965 (14 568.50‐96 163)	39 650 (18 034‐93 399)	<.001
Disposition of the patient at discharge			
Routine	15 738 (23.3%)	823 (22.7%)	<.001
Short‐term hospital	5960 (8.8%)	330 (9.1%)	
Other nursing care facilities	6822 (10.1%)	170 (4.7%)	
Home health care	2464 (3.7%)	41 (1.1%)	
Left against medical advice	214 (0.3%)	22 (0.6%)	
In‐hospital mortality	97 332 (54.7%)	6755 (61.7%)	<.001

Univariate predictors of in‐hospital mortality are demonstrated in Supplemental Table 2. Lack of health insurance (6.5% vs. 4.9%, *p* < .001), acute stroke (2.7% vs. 2.1%, *p* < .001), GI bleeding (5.3% vs. 2.7%, *p* < .001), AKI (28.2% vs. 17.3%, *p* < .001), cardiogenic shock (12.2% vs. 6.5%, *p* < .001), sepsis (6.3% vs. 3.8%, *p* < .001), mechanical ventilation (81.0% vs. 48.7%, *p* < .001), and low household income (46.9% vs. 41.6%, *p* < .001) were significantly associated with a higher in‐patient mortality. In‐hospital revascularization with either PCI or CABG surgery was associated with a significant reduction in in‐hospital mortality (1.3% vs. 8.5%, *p* < .001).

Table 3 depicts independent predictors of in‐hospital mortality in the study population. Lack of health insurance was independently associated with a higher in‐hospital mortality (1.53, 95% C.I. [1.46–1.61]; *p* < .001). Other independent predictors of in‐hospital mortality included GI bleeding (1.55, 95% C.I [1.46–1.64]; *p* < .001), AKI (1.20, 95% C.I [1.17–1.24]; *p* < .001), cardiogenic shock (1.51, 95% C.I [1.45–1.57]; *p* < .001), sepsis (1.12, 95% C.I [1.07–1.18]; *p* < .001), mechanical ventilation (4.17, 95% C.I [4.07–4.27]; *p* < .001), and Charlson comorbidity index (1.03, 95% C.I [1.03–1.04], *p* < .001). Revascularization (0.12, 95% C.I [0.12–0.13]; *p* < .001) was independently associated with a reduction in in‐hospital mortality. The multivariate model demonstrated good discrimination (ROC‐derived AUC = 0.72).

**TABLE 3:** Multivariate predictors of in‐hospital mortality

Independent variables	In‐hospital mortality	
	OR (95% CI)	*p*‐value
Lack of health insurance	1.53 (1.46–1.61)	<.001
In‐hospital revascularization	0.12 (0.11–0.13)	<.001
Mechanical ventilation	4.17 (4.07–4.27)	<.001
Acute cerebrovascular accident	2.87 (2.72–3.02)	.662
Acute kidney injury	1.21 (1.17–1.24)	<.001
Cardiogenic shock	1.51 (1.46–1.58)	<.001
Sepsis	1.12 (1.07–1.18)	<.001
Gastrointestinal bleeding	1.55 (1.46–1.64)	<.001
Charlson comorbidity index	1.03 (1.03–1.04)	<.001

Abbreviations: CI, confidence interval; OR, odds ratio.

Supplemental Table 3 describes univariate predictors of ICD utilization. ICD utilization was higher in younger patients and those with prior MI and previous or in‐hospital coronary revascularization. Female gender, lack of insurance, drug overdose, low income category, sepsis, dementia, coma, do not resuscitate status, cancer, discharge AMA status, and in‐hospital mortality were significantly associated with lower utilization of ICD.

Independent predictors of ICD utilization are demonstrated in Table 4. Lack of insurance was independently associated with a lower ICD utilization (0.84, 95% C.I [0.79–0.90], *p* < .001). Other independent predictors associated with lower ICD utilization were in‐hospital coronary revascularization (0.78, 95% C.I [0.74–0.82], *p* < .001), sepsis (0.78, 95% C.I [0.73–0.85], *p* < .001), coma (0.84, 95% C.I [0.81–0.86), *p* < .001), discharge AMA status (0.07, 95% C.I [0.05–0.10], *p* < .001), and in‐hospital mortality (0.004, 95% C.I [0.003–0.004], *p* < .001). The multivariate model demonstrated excellent statistical discrimination (ROC‐derived AUC = 0.87).

**TABLE 4:** Independent predictors of internal cardioverter defibrillator utilization in the study population

Variables	ICD Utilization	
	OR (95% CI)	*p*‐value
Lack of health insurance	0.84 (0.79–0.93)	<.001
In‐hospital revascularization	0.78 (0.74–0.82)	<.001
Sepsis	0.78 (0.73–0.84)	<.001
Coma	0.84 (0.81–0.86)	<.001
In‐hospital mortality	0.004 (0.003–0.004)	<.001
Left against medical advice status	0.07 (0.05–0.11)	<.001

Abbreviations: CI, confidence interval; ICD, internal cardioverter defibrillator; OR, odds ratio.

Univariate predictors of cost of hospitalization are depicted in Supplemental Table 4. Lack of insurance was associated with higher cost of hospitalization ($) (median [interquartile range], *p*‐value) (39 650 [18 034‐93 399] vs. 35 965 [14 568.50‐96 163], *p* < .001). Other univariate predictors of cost were in‐hospital coronary revascularization, mechanical ventilation, ICD insertion, and mechanical circulatory support (use of intraaortic balloon pump or left ventricular assist device) were associated with higher hospital cost. In‐hospital mortality and discharge AMA status were associated with lower cost of hospitalization. Table 5 demonstrates independent predictors of cost of hospitalization. In‐hospital coronary revascularization, ICD implantation, mechanical ventilation, mechanical circulatory support and longer length of stay were independently associated with increase in cost of hospitalization. Lack of insurance and discharge AMA status were not independently associated with a higher hospital cost.

**TABLE 5:** Independent predictors of cost of hospitalization

Variables	Cost of Hospitalization ($)		
	Mean	95% CI	*p*‐value
Lack of health insurance	−1229.1	−2856.9 – 398.7	.139
In‐hospital mortality	−13 649.6	−14 620.2 – −12 678.9	<.001
Length of stay	6160.7	6118.3–6203.2	<.001
ICD utilization	88 375.1	87 228.2–89 522.1	<.001
In‐hospital revascularization	56 330.3	54 505.4–58 155.3	<.001
Mechanical circulatory support	56 091.4	53 626.2–58 566.6	<.001
Mechanical ventilation	27 735	26 863.2–28 608.6	<.001
Left against medical advice status	−2874.2	6118.3–6203.2	.40

Abbreviations: CI, confidence interval; ICD, internal cardioverter defibrillator.

## 4 DISCUSSION

Our data show that in‐hospital mortality of uninsured patients presenting with out‐of‐hospital VF arrest is significantly higher compared to those with insurance. Uninsured patients were also significantly less likely to receive secondary prevention therapies such as internal cardioverter defibrillator implantation. The cost of care during the hospitalization was significantly higher in the uninsured cohort compared to those with insurance, likely driven by utilization of cost heavy services such as mechanical ventilation, and revascularization procedures.

Many plausible etiologies have been proposed for the outcome disadvantage observed in patients without insurance both in an outpatient setting as well as in the acute setting for conditions such as ST‐segment elevation myocardial infarction.^6^ Lack of continuity care follow‐up by primary care with ongoing preventative therapies and education might be responsible for this outcome disadvantage. Lack of diagnoses of chronic conditions amongst uninsured patients who may not access primary care may give a false impression of low comorbid complexity to the acute care team and such underdiagnoses may lead to a negative effect imparted by these unknown comorbidities on acute care outcomes. Underdiagnoses of chronic comorbidities may be responsible for the lower Charlson comorbidity score and a lower recorded incidence of key contributor comorbidities amongst uninsured patients in this analysis. Lack of insurance, which likely is associated with general lack of regular contact with the primary care medical system, may be associated with lack of chronic cardiac risk factor and cardiac disease awareness, poor understanding of the symptoms and signs of cardiac conditions, as well as related delays in seeking appropriate and timely medical care.

Uninsured status was independently associated with a decrease in utilization of implantable cardioverter defibrillator therapy after an OHVFA. This difference persisted after adjusting for contributory factors such as leaving against medical advice, advanced directives, and neurologic compromise. The cause and appropriateness of this decreased utilization of ICD implantation in uninsured patients is unclear from our dataset but deserves further assessment.

The cost of hospital care for uninsured patients was significantly higher compared to that insured counterparts. This difference was not seen after adjusting for some of the other contributors to the cost of care such as intensive care therapies including mechanical ventilation, and need for mechanical circulatory support which were higher in uninsured patients suggesting these to be the mediators of higher unadjusted cost of hospitalization seen in the uninsured patients. Higher utilization of these treatments could well be a reflection of the unknown comorbid complexity of uninsured patients.

The higher incidence of behaviors such as history of drug abuse, as well as a higher prevalence of leaving against medical advice, imply a lower level of health awareness and care engagement amongst the uninsured cohort. Healthcare policy interventions to address socioeconomic determinants of health, improving access to health insurance. Enhancing patient education and engagement in both primary care and hospital settings may have potential for improving outcomes of catastrophic presentations such as OHVFA.

## 5 LIMITATIONS

Our observational study from the nation's largest all‐payer administrative hospital database has limitations and biases. Being an administrative database, the NIS is subject to coding errors and residual confounding exists. Lack of procedural, as well as laboratory and pharmacotherapy data, further limit the analyses. Since NIS records individual hospitalizations, long‐term outcomes impact of insurance status on out‐of‐hospital VF arrest cannot be analyzed. Our analyses are limited to patients with OHVFA and hence should not be extrapolated to cardiac arrests associated with asystole or PEA.

## 6 CONCLUSIONS

Uninsured victims of out‐of‐hospital VF arrest have a higher adjusted in‐hospital mortality compared to those with health insurance.

## Supporting information


**Supplemental Table 1** International Classification of Disease, Ninth Revision, Clinical Modification (ICD‐9‐CM) and Clinical Classification Software (CCS) Codes Used to Identify Comorbidities

**Supplementary Table 2** Univariate Predictors of In‐hospital Mortality in the Study Population

**Supplementary Table 3** Univariate Predictors of Implantable Cardioverter Defibrillator Utilization in the Study Population

**Supplementary Table 4** Univariate Predictors of Cost of Hospitalization in the Study Population

## Data Availability

The data that support the findings of this study are available from Health Care Utilization Project. Restrictions apply to the availability of these data, which were used under license for this study. Data are available with the permission of Health Care Utilization Project.
