# Supplementary material for: Association between insurance status and in‐hospital outcomes in patients with out‐of‐hospital ventricular fibrillation arrest
Source: Clin Cardiol. 2021 Mar 4;44(4):511–7. doi: 10.1002/clc.23564 (PMC8027577; doi:10.1002/clc.23564)
Supplement: Supplementary file 1 — Supplemental Table 1 International Classification of Disease, Ninth Revision, Clinical Modification (ICD‐9‐CM) and Clinical Classification Software (CCS) Codes Used to Identify Comorbidities [file CLC-44-511-s003.docx]

**Supplemental Table 1. International Classification of Disease, Ninth Revision, Clinical Modification (ICD-9-CM) and Clinical Classification Software (CCS) Codes Used to Identify Comorbidities**

| **Comorbidities** | **ICD-9-CM Codes** | **CCS Codes** |
| --- | --- | --- |
| Ventricular fibrillation | 427.4, 427.41, 427.42, 427.5 |  |
| Acute cerebrovascular accident |  | 109 |
| Prior stroke |  | 113 |
| Diabetes mellitus |  |  |
| Without end-organ damage | 250-250.33 |  |
| With end-organ damage | 250.4-250.93 |  |
| Hypertension |  |  |
| Without complications |  | 98 |
| With complications |  | 99 |
| Congestive heart failure | 398.91, 402.01, 402.11, 402.91, 404.01, 404.11, 404.91, 428.0, 428.1, 428.2, 428.20, 428.21, 428.22, 428.23, 428.3, 428.30, 428.31, 428.32, 428.33, 428.4, 428.40, 428.41, 428.42, 428.43, 428.9 |  |
| Peripheral vascular disease | 440, 440.0, 440.1, 440.2, 440.20, 440.21, 440.22, 440.23, 440.24, 440.29, 440.3, 440.30, 440.31, 440.32, 440.4, 440.8, 440.9, 441, 441.0, 441.00, 441.01, 440.02, 441.03, 441.1, 441.2, 441.3, 441.4, 441.5, 441.6, 441.7, 441.9, 442, 442.0, 442.1, 442.2, 442.3, 442.8, 442.81, 442.82, 442.83, 442.84, 442.89, 442.9, 443, 443.0, 443.1, 443.2, 443.21, 443.22, 443.23, 443.24, 443.29, 443.8, 443.81, 443.82, 443.89, 443.9, 447.1, 557.1, 557.9 |  |
| Chronic kidney disease |  | 158 |
| Sepsis | 995.91, 785.52, 995.92 |  |
| Cardiogenic shock | 785.51 |  |
| Mechanical circulatory support |  |  |
| Intraaortic balloon pump | 37.61 |  |
| Left ventricular assist device | 37.68 |  |
| Percutaneous coronary intervention |  | 45 |
| Coronary artery bypass graft surgery | 36.10, 36.11,  36.12, 36.13, 36.14, 36.15 |  |
| Atrial fibrillation | 427.31 |  |
| Atrial flutter | 427.32 |  |
| Gastrointestinal bleeding |  | 153 |
| Acute kidney injury |  | 157 |
| Smoking | 305.1, V15.82 |  |
| Alcoholism | 305.0, 305.00, 305.01, 305.02, 305.03, 303, 303.0, 303.00, 303.01, 303.02, 303.03, 303.9, 303.90, 303.91, 303.92, 303.93 |  |
| Drug abuse | 305.x |  |
| Previous myocardial infarction | 412 |  |
| Previous coronary artery bypass graft surgery | V45.81 |  |
| Previous percutaneous coronary intervention | V45.82 |  |
| Valvular heart disease | 394, 394.0, 394.1, 394.2, 394.9, 395, 395.0, 395.1, 395.2, 395.9, 396, 396.0, 396.1, 396.2, 396.3, 396.8, 396.9 |  |
| Long-term use of anticoagulants | V58.61 |  |
| Body mass index 25-29.9 kg/m^2^ | 278.02 |  |
| Body mass index 30-39.9 kg/m^2^ | 278.00 |  |
| Body mass index ≥ 40 kg/m^2^ | 278.01 |  |
| Mechanical ventilation | 96.70, 96.71, 96.72 |  |
| Internal cardioverter defibrillation | 37.94, 37.95, 00.51 |  |
| Do not resuscitate status | V49.86 |  |
| Coma |  | 86 |
| Cancer |  | 11, 12, 13, 14, 15, 16, 17, 18, 19, 20, 21, 22, 23, 24, 25, 26, 27, 28, 29, 30, 31, 32, 33, 34, 35, 36, 37, 38, 39, 40, 41, 42, 43, 44, 46, 47 |
