# Supplementary material for: Association between insurance status and in‐hospital outcomes in patients with out‐of‐hospital ventricular fibrillation arrest
Source: Clin Cardiol. 2021 Mar 4;44(4):511–7. doi: 10.1002/clc.23564 (PMC8027577; doi:10.1002/clc.23564)
Supplement: Supplementary file 2 — Supplementary Table 2 Univariate Predictors of In‐hospital Mortality in the Study Population [file CLC-44-511-s004.docx]

**Supplementary Table 2. Univariate Predictors of In-hospital Mortality in the Study Population**

| **Variables** | **Survived**  **(n=84,859)** | **Died**  **(n=104,087)** | **p-value** |
| --- | --- | --- | --- |
| In-hospital revascularization | 7186 (8.5%) | 1357 (1.3%) | <0.001 |
| Acute cerebrovascular accident | 1776 (2.1%) | 2789 (2.7%) | <0.001 |
| Gastrointestinal bleeding | 2278 (2.7%) | 5487 (5.3%) | <0.001 |
| Acute kidney injury | 14,690 (17.3%) | 29,323 (28.2%) | <0.001 |
| Cardiogenic shock | 5534 (6.5%) | 12,716 (12.2%) | <0.001 |
| Sepsis | 3256 (3.8%) | 6520 (6.3%) | <0.001 |
| Mechanical Ventilation | 41,317 (48.7%) | 84,280 (81%) | <0.001 |
| Charlson comorbidity index [Median (interquartile range)] | 2.0 (1.0-3.0) | 2.0 (1.0-4.0) | <0.001 |
| Lack of health insurance | 4186 (4.9%) | 6755 (6.5%) | <0.001 |
| Median household income category^a^ |  |  |  |
| Below median national income category | 45,097 (53.1%) | 39,762 (46.9%) | <0.001 |
| Above median national income category | 60,781 (58.4%) | 43,306 (41.6%) |  |

^a^This represents a quartile classification of the estimated median household income of residents in the patient’s zip code.
