# Supplementary material for: Association between insurance status and in‐hospital outcomes in patients with out‐of‐hospital ventricular fibrillation arrest
Source: Clin Cardiol. 2021 Mar 4;44(4):511–7. doi: 10.1002/clc.23564 (PMC8027577; doi:10.1002/clc.23564)
Supplement: Supplementary file 3 — Supplementary Table 3 Univariate Predictors of Implantable Cardioverter Defibrillator Utilization in the Study Population [file CLC-44-511-s002.docx]

**Supplementary Table 3. Univariate Predictors** **of Implantable Cardioverter Defibrillator Utilization in the Study Population**

| **Characteristics** | **No ICD**  **(n=154,755)** | **ICD**  **(n=34,191)** | **p-value** |
| --- | --- | --- | --- |
| Age (Years) [Median (interquartile range)] | 68.0 (57.0-79.0) | 61.0 (52.0-71.0) | <0.001 |
| Male | 88,238 (57.0%) | 23,025 (67.4%) | <0.001 |
| Female | 66,465 (43.0%) | 11,151 (32.6%) |  |
| Median household income category^a^ |  |  |  |
| Below median national income category | 88,738 (57.3%) | 17,140 (50.1%) | <0.001 |
| Above median national income category | 66,017 (42.7%) | 17,051 (49.9%) |  |
| Sepsis | 8699 (5.6%) | 1077 (3.1%) | <0.001 |
| Previous myocardial infarction | 13,357 (8.6%) | 5551 (16.2%) | <0.001 |
| Prior revascularization | 20,167 (13.0%) | 6822 (20.0%) | <0.001 |
| Dementia | 5232 (3.4%) | 235 (0.7%) | <0.001 |
| Coma | 76046 (49.1%) | 9393 (27.5%) | <0.001 |
| Cancer | 18,678 (12.1%) | 2747 (8.0%) | <0.001 |
| Drug abuse | 1008 (0.7%) | 82 (0.2%) | <0.001 |
| Left against medical advice | 559 (0.4%) | 33 (0.1%) | <0.001 |
| Do Not Resuscitate status | 16,372 (10.6%) | 108 (0.3%) | <0.001 |
| In-hospital revascularization | 5980 (3.9%) | 2563 (7.5%) | <0.001 |
| In-hospital mortality | 103,867 (67.1%) | 220 (0.6%) | <0.001 |
| Lack of health insurance | 9,269 (6.0%) | 1,672 (4.9%) | <0.001 |

Abbreviations: ICD, Internal cardioverter defibrillator.

^a^This represents a quartile classification of the estimated median household income of residents in the patient’s zip code.
