# Supplementary material for: Association between insurance status and in‐hospital outcomes in patients with out‐of‐hospital ventricular fibrillation arrest
Source: Clin Cardiol. 2021 Mar 4;44(4):511–7. doi: 10.1002/clc.23564 (PMC8027577; doi:10.1002/clc.23564)
Supplement: Supplementary file 4 — Supplementary Table 4 Univariate Predictors of Cost of Hospitalization in the Study Population [file CLC-44-511-s001.docx]

**Supplementary Table 4. Univariate Predictors** **of Cost of Hospitalization in the Study Population**

| **Characteristics** | **Cost of Hospitalization ($)**  **[Median (interquartile range)]** | **p-value** |
| --- | --- | --- |
| Lack of health insurance | 39,650 (18,034-93,399) | <0.001 |
| In-hospital mortality | 22,048 (11,176-45,874) | <0.001 |
| In-hospital revascularization | 135,287 (82,218-225,881) | <0.001 |
| ICD utilization | 141,805 (97,732-211,630) | <0.001 |
| Mechanical Ventilation | 37,913 (17,183-97,792) | <0.001 |
| Mechanical circulatory support | 106,323 (55,921-198,900) | <0.001 |
| Left against medical advice status | 33,931 (13,059-74,170) | <0.001 |

Abbreviations: ICD, Internal cardioverter defibrillator.
